# Functional Overview of Plant Genes Essential for Arbuscular Mycorrhizal Symbiosis

**DOI:** 10.3390/genes17060686

**Published:** 2026-06-11

**Authors:** Shang Wang, Jiali Yi, Zheyu Li, Jiayao Wu, Yufeng Xu, Runhan Xue, Yiang Wang, Lihui Duan, Likang Zhao, Erxu Pi

**Affiliations:** 1 College of Life and Environmental Sciences, Hangzhou Normal University, Hangzhou 311121, China; 2 Zhejiang Province Key Laboratory of Plant Secondary Metabolism and Regulation, Zhejiang Sci-Tech University, Hangzhou 310018, China

**Keywords:** AM symbiosis, arbuscular mycorrhizal fungus (AMF), symbiosis-essential genes, strigolactones (SLs), arbuscule development

## Abstract

Since the first plant gene essential for arbuscular mycorrhizal (AM) symbiosis was identified, more than 170 such genes have been discovered. However, these findings remain scattered across species, and a systematic synthesis is urgently needed to guide future functional studies and breeding applications. This review provides a systematic classification and contextual overview of the currently reported plant genes essential for AM symbiosis, covering leguminous species (e.g., *Medicago truncatula, Lotus japonicus*) and non-leguminous species (e.g., *Oryza sativa*, *Solanum lycopersicum*). We classify these genes into functional modules corresponding to key stages of AM symbiosis: SL-mediated pre-symbiotic signaling, chitin signal perception, activation of the common symbiosis signaling pathway (CSSP), calcium (Ca^2+^) oscillation generation, arbuscule development and maintenance, and nutrient exchange. Beyond classification, we highlight conserved genetic modules across plant lineages and discuss their implications for engineering AM symbiosis in non-host or poorly symbiotic crops. This synthesis establishes a foundational genetic resource for molecular breeding aimed at enhancing nutrient-use efficiency and sustainable crop production.

## 1. Introduction

As the oldest and most widespread mutualistic symbiosis between terrestrial plants and fungi of the phylum Glomeromycota, AM symbiosis originated approximately 460 million years ago, coinciding with the emergence of the earliest land plants. By providing a critical interface for nutrient acquisition, this symbiosis has significantly facilitated the transition of plants from aquatic to terrestrial life. Among contemporary plants, approximately 80% of terrestrial plant species, including those from the Fabaceae (e.g., *M. truncatula*, *L. japonicus*), Poaceae (e.g., *O. sativa*, *Zea mays*), and Solanaceae (e.g., *S. lycopersicum*), are capable of establishing symbiotic associations with AMF. In contrast, Brassicaceae (including *Arabidopsis thaliana*) lack the capacity to form such interactions [1,2,3,4].

During the establishment of symbiosis, plant roots release SLs into the rhizosphere, which act as signaling molecules to stimulate hyphal branching of AMF. In turn, the fungi release diffusible signals such as chitin oligosaccharides (Myc-LCOs/COs), which are perceived by root epidermal cells. This recognition process activates a response that depends on the CSSP, involving components such as nucleoporins and nuclear membrane-localized cation channels. These events ultimately induce Ca^2+^ oscillations in the nucleus of host root cells. These Ca^2+^ oscillations are sensed by a calcium- and calmodulin-dependent protein kinase (CCaMK), which subsequently activates downstream transcription factors [3,5,6].

**Figure 1:**
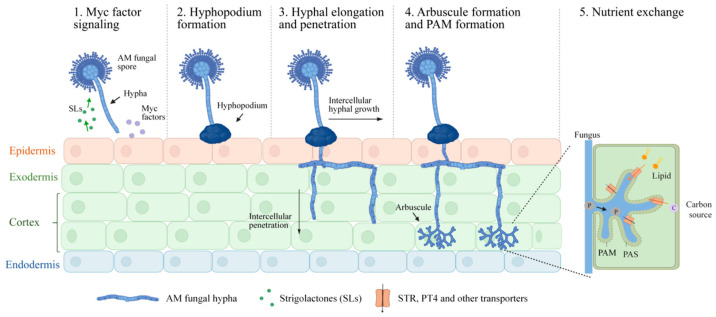
Overview of arbuscular fungal dynamics and arbuscule formation. This schematic diagram shows the continuous whole process of AMF colonization and arbuscule formation in plant roots. The root tissues are stratified into four layers (epidermis, exodermis, cortex, and endodermis), featuring an exodermal suberin barrier and gradual physiological variations among tissue layers. The entire symbiotic process consists of five sequential core stages: (1) Myc factor signaling. Plant root cells perceive fungal signaling molecules (e.g., LCOs/COs) to initiate the AMF–plant symbiotic interaction. (2) Hyphopodium formation. Upon initial physical contact with root epidermal cells, AMFs form a specialized infection structure termed the hyphopodium on the root surface. (3) Hyphal elongation and penetration. Fungal hyphae elongate from hyphopodia, penetrate the epidermal cell layer, and further grow intra- and intercellularly across the exodermis and outer cortex, ultimately progressing toward the inner cortical layers. (4) Arbuscule and PAM formation. Fungal hyphae invade inner cortical cells and undergo extensive branching to form intracellular arbuscules. (5) Nutrient exchange. The highly branched arbuscules act as the key site for bidirectional material exchange. AMFs provide inorganic phosphate (Pi) and other mineral nutrients to host plants and acquire organic carbon and lipids from plant cells in return.

Once AMF hyphae penetrate the epidermis and enter root cortical cells, the root cortical cells undergo extensive transcriptional reprogramming to accommodate the symbiont. Within the cortical cells, the fungus develops highly branched arbusculars, which are enveloped by the plant-derived periarbuscular membrane (PAM) [7,8]. The PAM harbors phosphate, ammonium, nitrate, and lipid transporters (e.g., STR), which are responsible for the efficient transfer of nutrients such as phosphorus and nitrogen from the fungus to the plant. In exchange, the plant delivers up to 20% of its fixed carbon and essential fatty acids to the obligate biotrophic fungus via sugar transporters (SUTs, SWEETs) and the lipid transfer system (Figure 1) [2,6,9,10].

Since the first gene related to AM symbiosis was identified in legumes, systematic progress has been made in understanding the molecular mechanisms underlying this symbiosis. Subsequently, research on AM symbiosis in non-legumes has also entered a period of rapid development, greatly expanding our understanding of the conserved symbiotic mechanisms across different plant lineages. In mycorrhizal host plants, loss-of-function screening is often time-consuming and challenging due to genetic redundancy, resulting in many key regulatory factors, including transcription factors, remaining to be fully characterized [11].

**Figure 2:**
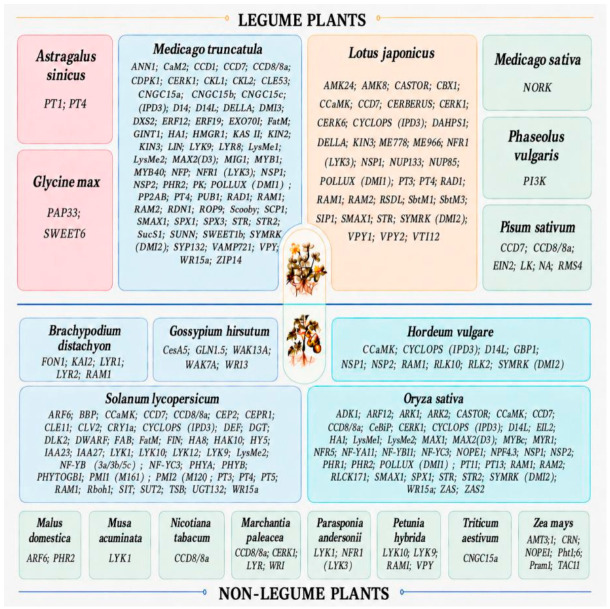
Classification and species distribution of AM symbiosis-related plant genes included in this study: A total of 172 functionally validated plant genes involved in AM symbiosis were compiled in this study. Based on the evolutionary status and research background of the species, these genes were classified into two major groups-legumes and non-legumes—and were systematically categorized and summarized by species.

Based on this, the present review systematically summarizes AM symbiosis-related genes identified over the past three decades, compiling a total of 172 functionally validated genes (Table S1). These genes were classified according to the evolutionary status and research background of the species. They are primarily derived from legumes (e.g., *M. truncatula*, *L. japonicus*, *Glycine max*) and non-legumes (e.g., *O. sativa*, *S. lycopersicum*, *Z. mays*, *Brachypodium distachyon*) and also include orthologs identified in basal land plants (e.g., *Marchantia paleacea*). Based on the above classification, we constructed a species distribution map of AM symbiosis-related genes, illustrating the number and types of identified genes across different plant lineages (Figure 2).

Legumes (Fabaceae) include the following species: *L. japonicus*, *Glycine max*, *Phaseolus vulgaris*, *Pisum sativum*, *M. sativa*, *M. truncatula*, *Astragalus sinicus*. Non-legumes include the following species: *B. distachyon*, *Gossypium hirsutum*, *Hordeum vulgare*, *Malus domestica*, *M. paleacea*, *Musa acuminata*, *Nicotiana tabacum*, *O. sativa*, *Parasponia andersonii*, *Petunia hybrida*, *S. lycopersicum*, *Triticum aestivum*, *Z. mays.* The classification diagram intuitively displays the distribution of gene numbers across different plant lineages. See also Table S1 for detailed data.

**Figure 3:**
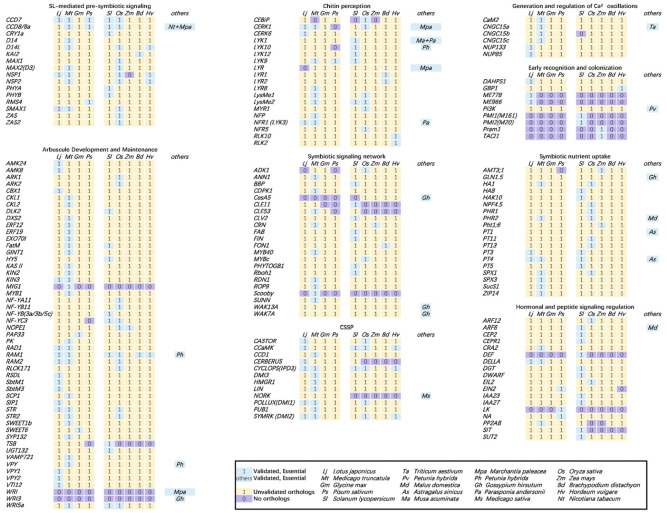
Presence and functional requirements of AM symbiosis-related genes across different plant species. The presence or absence of orthologous genes is indicated by “1” (present) or “0” (absent). Blue (1) and others: experimentally validated AM symbiosis genes; yellow (1): orthologs present but AM symbiosis function unverified; purple (0): no ortholog identified. Data are based on the published literature, where available, and sequence information from public data repositories. These analyzed species include four legumes and five non-legumes. An additional column “Others” indicates that the gene has also been functionally studied in additional species beyond these nine representatives. See also Table S1.

To investigate the evolutionary conservation and functional divergence of AM symbiosis-related genes across the plant kingdom, we selected nine representative species, covering the Fabaceae (including *L. japonicus*, *M. truncatula*, *G. max*, *P. sativum*), Solanaceae (*S. lycopersicum*), and Poaceae (*O. sativa*, *Z. mays*, *B. distachyon*, *H. vulgare*). These species possess well-annotated genomes and rich genetic resources and have accumulated extensive functional validation data in the field of AM symbiosis, providing a solid foundation for cross-species orthologous comparison. Based on the sequences of functionally validated genes, we performed systematic orthologous screening and comparative analysis. Subsequently, focusing on key steps in AM symbiosis establishment, including SL-mediated pre-symbiotic signaling, chitin signal perception, CSSP, Ca^2+^ spiking generation and decoding, early signal recognition and regulation, arbuscule development and maintenance, and symbiotic nutrient uptake, we summarized the presence (Figure 3), sequence features, and potential functional conservation of these genes across different species (Table S1). This analysis provides an important basis for understanding the evolutionary trajectory and species-specific adaptations of AM symbiosis. These findings not only deepen our understanding of the molecular network underlying plant–fungal mutualism but also provide important theoretical foundations and genetic resources for future genetic improvement aimed at enhancing crop nutrient use efficiency and agricultural sustainability.

## 2. SL-Mediated Pre-Symbiotic Signaling

SLs are multifunctional carotenoid-derived plant metabolites with diverse biological functions in plant growth and development. Crucially, root-secreted SLs act as core rhizosphere signaling molecules governing pre-symbiotic interactions with AM fungi. Although SL biosynthesis and signaling are not uniquely dedicated to symbiosis, the conserved SL synthetic and transduction machinery is specifically required for triggering early pre-symbiotic communication and facilitating AM symbiosis establishment under phosphate starvation.

### 2.1. Key SL Biosynthetic Enzymes Associated with AM Pre-Symbiotic Signaling

SL biosynthesis requires multiple enzymes, among which CCD7 (carotenoid cleavage dioxygenases) and CCD8 act as core rate-limiting components. These two enzymes are indispensable for SL-mediated pre-symbiotic signaling and AM symbiosis, with conserved but species-diversified regulatory functions.

Defective CCD7 impairs mycorrhizal colonization in *M. truncatula* [12] and tomato [13]. Furthermore, the *ccd7* mutant displays defects in red-light-regulated AM symbiosis and phosphorus accumulation [14], whereas *L. japonicus* can maintain normal AM colonization despite reduced SL levels after LjCCD7 silencing, suggesting species-specific regulatory redundancy [15]. By contrast, CCD8 primarily supports early symbiosis establishment [16]. Reduced hyphal branching and impaired mycorrhizal colonization are observed in pea *ccd7* and *ccd8* mutants [17]. Mutations in tobacco [18] and *M. truncatula* [19] CCD8 consistently cause SL deficiency, defective fungal hyphal branching, and reduced colonization. In rice, the CCD8 homolog D10 is required for fungal appressorium formation and sustained root colonization [20,21]. Evolutionarily, CCD8-mediated SL synthesis represents an ancestral symbiotic mechanism, as redundant MpCCD8A/8B in the bryophyte *M. paleacea* ensures basal SL production and ancient AM symbiosis [22].

In addition, other CCD family members also modulate AM symbiosis by regulating apocarotenoid and SL metabolism. Rice *OsZAS* and *OsZAS2* encode zaxinone synthases; the mutation disrupts SL biosynthesis and thereby reduces AM colonization [23,24]. MtCCD1 suppression disrupts symbiosis-related apocarotenoid metabolism and mildly affects arbuscule development [25].

### 2.2. Functional Diversification of SL Receptors in Regulating AM Symbiosis

Dwarf14 (D14) is a well-characterized receptor for SLs [26]. By contrast, Dwarf14-Like (D14L), an α/β hydrolase homologous to D14, acts as a receptor for a broader range of butenolides, including both SLs and karrikin-like molecules [12], and has been demonstrated to be essential for the establishment of AMF colonization in rice [27,28], *M. truncatula* and *H. vulgare* [12]. Under nutrient limitation, NSP1/NSP2 coordinate SL biosynthesis and chitin perception to amplify pre-symbiotic responses [12]. Accessories, including KAI2 [29], D3/MAX2 [20,26,27], and OsMAX1 [30] family proteins, further support D14L-dependent symbiotic signaling.

### 2.3. Regulatory Network and Adaptive Plasticity of SL Symbiotic Signaling

SL-mediated pre-symbiotic signaling is precisely modulated by endogenous and environmental cues to balance symbiotic performance and plant growth. The D14L signaling cascade activates AM symbiosis by promoting the degradation of the repressor SMAX1, while ethylene stabilizes SMAX1 protein to suppress mycorrhizal colonization [12,26,31]. Light signaling serves as a key environmental regulator: the phyB/cry1a-HY5 module transcriptionally upregulates CCD7, CCD8, and MAX1 to promote SL synthesis and symbiosis, while far-red light exerts negligible effects [14]. Multiple upstream regulators, including RMS4O [32], ORT1 [33], RMS1 [34], NSP1 and NSP2, further link nutrient status to SL signaling and fine-tune AM colonization efficiency [33,35]. Furthermore, NSP1 in *M. truncatula*, *L. japonicus*, and *H. vulgare*, as well as NSP2 in *M. truncatula* and *H. vulgare*, positively regulate AM colonization, with their mutants exhibiting significantly reduced AM colonization levels [12,33,35,36].

SL-mediated pre-symbiotic signaling exhibits conserved core mechanisms and flexible regulatory plasticity to sustain stable AM symbiosis. The core SL biosynthesis and D14L-dependent signaling pathways are evolutionarily conserved in land plants, and disruption of key genes severely impairs early mycorrhizal colonization. Meanwhile, this symbiotic pathway possesses remarkable functional redundancy and environmental adaptability across plant species and growth stages. Multiple compensatory mechanisms support symbiotic robustness, including temporal colonization recovery in rice d10 mutants [37], functional redundancy of LjCCD7 and MpCCD8a/8b [15,22], and unchanged symbiotic performance in sorghum lgs1 mutants with altered SL components [38]. Additionally, symbiosis-induced blumenols can partially compensate for impaired SL signaling to maintain symbiotic homeostasis [12]. Collectively, these layered conservation and compensatory mechanisms enable flexible plant–AM fungus interactions, offering valuable genetic targets for improving crop nutrient utilization efficiency.

## 3. Chitin Perception

AM fungi activate symbiosis through chitin-derived signals (LCOs and COs). Plant perception of these signals is not a simple on-off switch but a multi-layer, cell-surface signaling gateway that integrates ligand discrimination, immune suppression, and nutrient status to initiate the CSSP.

**Figure 4:**
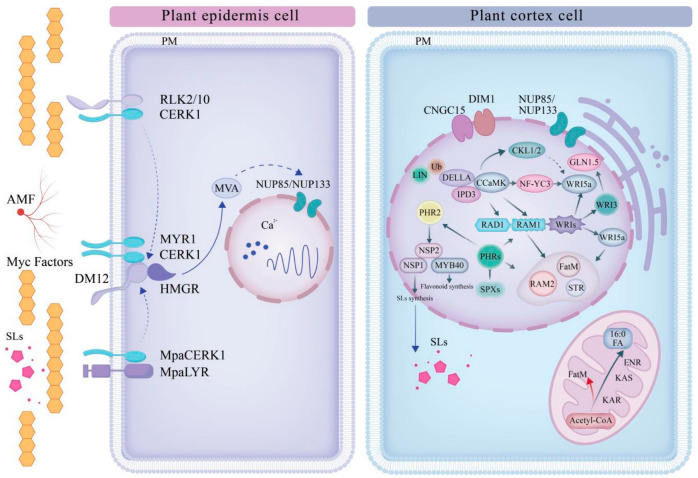
A working model of AM symbiosis signaling and cellular remodeling, with genes and regulatory pathways involved in AM symbiosis signaling and cellular remodeling under low-phosphate conditions. This schematic diagram displays the complete AM symbiosis signaling process via juxtaposed epidermal and cortical cell modules. Under phosphate deficiency, plant roots secrete SLs to stimulate AM fungi to produce Myc factors. The generated Myc factors are perceived by root epidermal receptors (e.g., MYR1, CERK1), triggering Ca^2+^ spiking and the activation of the CSSP. The activated symbiotic signals propagate inward and trigger nuclear responses in cortical cells, modulating multiple transcription factors and inducing the expression of symbiotic genes. The core regulator RAM1 initiates downstream lipid biosynthesis, which provides essential materials for PAM formation. Key proteins involved [e.g., DMI2, CCaMK, CYCLOPS, DELLA, WRI5a, RAM1, RAM2, FatM, PHR2, VAPYRIN(VPY)] are indicated. See main text for details. It should be noted that the functional partitioning between cell layers is not absolute. Some processes may operate in both cell types but potentially serve distinct functions. The cellular model presented here serves as a reference to illustrate the predominant spatial distribution of symbiotic events based on current evidence, rather than a strict delineation of cell-type-specific exclusivity.

At the plasma membrane of epidermal cells, distinct LysM-RLK heterodimers discriminate between different chitinous ligands. In rice, the CO4-specific receptor OsMYR1 pairs with the dual-function kinase OsCERK1 (required for both symbiosis and immunity) to activate CSSP [39,40,41]. OsCEBiP is essential for longer CO8 perception; its loss reduces CO8 response but unexpectedly increases early colonization, indicating that dampening immune detection facilitates fungal entry [39,42]. In legumes, NFR1/LYK3 specifically perceives Myc-LCOs [40], while SlLYK1 acts as the key receptor responsible for long-chain chitin CO8 perception [43], and in barley, the NFR5 homolog HvRLK10 cooperates with RLK2 for LCO perception [12]. This receptor logic is deeply conserved: even the liverwort *M. paleacea* employs the ancient MpaCERK1-MpaLYR pair for chitin perception and AM symbiosis (Figure 4) [1].

Several LysM-RLKs have evolved bifunctional roles that help balance colonization with defense. Although a previous study reported that the *lys6* mutant had no significant effect on mycorrhizal colonization [44], further analysis revealed that LYS6/CERK6 in *L. japonicus* recognizes long-chain chitin to trigger defense responses, positively regulates mycorrhizal colonization, and plays an important role in CO4/CO8-induced Ca^2+^ signaling [45,46]. Similarly, *M. truncatula* LYK9 plays a dual role in disease resistance and AM symbiosis [47]. LYR-IB group receptors (e.g., MtLYR8) exhibit high affinity for LCOs and both short- and long-chain COs; their mutations specifically impair AM symbiosis without affecting rhizobial nodulation [48].

Beyond membrane-bound receptors, secreted LysM proteins fine-tune the symbiotic dialog at the interface. LysMe1/2 are conserved apoplastic proteins localized to arbuscule-containing cells, and mutations in *M. truncatula*, tomato, and rice severely reduce AMF colonization and arbuscule formation [19,49,50]. Ligand perception does not operate in isolation; it is tightly integrated with plant nutritional status. NSP1 and NSP2 regulate LCO perception under nutrient starvation; their double mutation markedly attenuates Ca^2+^ oscillations. Furthermore, D14L plays a key role in LCO perception and AMF colonization [12].

## 4. Generation and Regulation of Ca^2+^ Oscillations

Nuclear Ca^2+^ oscillations are key events in symbiotic signal transduction, facilitating plant recognition of AMF. DMI1 and CNGC15 play essential roles in symbiotic Ca^2+^ oscillations [51]; functional mutations in either gene severely impair Ca^2+^ spiking. DMI1 and CNGC15 form a complex at the nuclear envelope to coordinate these oscillations. The gain-of-function mutation of CNGC15 (CNGC15GoF) induces spontaneous low-frequency Ca^2+^ oscillations and, in concert with DMI1, enables Nod factors to trigger higher-frequency oscillations, thereby significantly enhancing AM colonization in alfalfa and wheat [52].

In contrast, holo-CaM2 (the calcium-bound form of CaM2) interacts with CNGC15 and provides negative feedback to close the channel, thereby regulating nuclear Ca^2+^ oscillations. Engineered holo-CaM2 accelerates Ca^2+^ oscillation frequency and early symbiotic signaling, enhancing root nodule symbiosis but not AM symbiosis [53].

NUP85 and NUP133 are nucleoporins essential for Ca^2+^ oscillations in the common symbiosis signaling pathway. Mutations in either gene lead to mycorrhizal colonization defects: in the *nup85* mutant, hyphae fail to penetrate the cortex; in both *nup85* and *nup133* mutants, Nod factors fail to induce Ca^2+^ spiking [54,55].

### Early Recognition and Colonization

During the early recognition and invasion stage of symbiosis, *Nope1* encodes an N-acetylglucosamine transporter belonging to the MFS superfamily. Its loss of function results in failure of appressorium formation in maize or formation of aberrant hyphopodia in rice, indicating its involvement in pre-contact early recognition [56]. In the *Taci1* mutant, appressorium formation frequency is reduced, but morphology is normal, and hyphae can penetrate the epidermis; however, after entering the root, most hyphae become septated, leading to termination of fungal spread. Pram1 exhibits the opposite phenotype: earlier and enhanced fungal invasion with normal hyphal morphology, indicating that it acts as a negative regulator of symbiosis [57].

During the nutrient exchange and arbuscule formation stage, DAHPS1 is the first enzyme of the shikimate pathway; its mutants exhibit delayed AM colonization and a reduced arbuscule formation rate [58]. PI3K is essential for root hair growth and curling, infection thread migration, nodulation, and AM symbiosis; its loss of function severely impairs root hair growth and curling, and AM fungi fail to enter epidermal and cortical cells, resulting in failure to form typical arbuscules and severely impaired colonization [59].

Regarding host defense regulation, barley GBP1 and GBP2 are GH81-type β-1,3-endoglucanases that regulate host defense by recognizing fungal β-glucans; mutations in both genes impair beneficial fungal colonization and lead to excessive activation of cell wall defense responses [60]. The tomato pmi mutants M161 and M20 both suppress AM fungal proliferation through root exudates, with M20 exhibiting a stronger inhibitory Myc^−^ phenotype than M161 [61,62].

## 5. CSSP

The CSSP is a core signaling module shared by plants for establishing symbiosis with both rhizobia and AMF. Its key components were initially identified in *M. truncatula* through “Nod-Myc” mutants, which are defective in both nodulation and mycorrhizal colonization.

### 5.1. Upstream Signal Perception and Initiation of Ca^2+^ Spiking

DMI1 and DMI2 are upstream components of the CSSP that together mediate periodic nuclear Ca^2+^ spiking upon symbiotic signal perception. Both Nod factors from rhizobia and hyphopodium exudates from AM fungi require DMI1 and DMI2 to induce nuclear Ca^2+^ spiking [63]. In *M. truncatula dmi1* and *dmi2* mutants, AM fungal exudates fail to induce nuclear Ca^2+^ oscillations, and the mutants are defective in both nodulation and mycorrhization [64,65,66]. Notably, although Ca^2+^ spiking is abolished, *dmi1* and *dmi2* mutants still exhibit Nod-factor-induced rapid Ca^2+^ influx [63].

HMGR1, a key regulatory enzyme of the mevalonate (MVA) pathway, catalyzes the conversion of HMG-CoA to MVA, interacts with DMI2, and is essential for initiating Ca^2+^ oscillations and symbiotic gene expression, indicating a critical role for the MVA pathway in early symbiotic signaling [67]. PUB1 is an E3 ubiquitin ligase that interacts with DMI2 and negatively regulates AM fungal infection and colonization through its ubiquitination activity [68].

### 5.2. Ca^2+^ Signal Decoding and Downstream Responses

DMI3 plays a dual role in both nodulation and mycorrhizal symbiosis. Unlike DMI1 and DMI2, the *dmi3* mutant can still generate Ca^2+^ spiking, indicating that DMI3 is not involved in Ca^2+^ spiking generation but rather in “reading” and “decoding” these Ca^2+^ signals [63,64,69]. DMI3/CCaMK act downstream of Ca^2+^ spiking, physically interact with the active kinase CYCLOPS, and phosphorylate CYCLOPS in vitro. Together, they coordinately regulate intracellular accommodation. CYCLOPS, a regulator of AM symbiosis, directly blocks arbuscular mycorrhiza formation when mutated in rice, *H. vulgare*, and *L. japonicus* [12,70,71]. Consistently, loss-of-function mutations of CCaMK in *H. vulgare*, *L. japonicus*, and *S. lycopersicum* all lead to defects in AM symbiosis [12,72,73]. In *M. truncatula*, *ipd3* and *ipd3l* mutants exhibit reduced mycorrhizal colonization [74,75]. In rice, *ipd3* mutation blocks AM symbiosis establishment, and the transcript of the mycorrhiza-specific phosphate transporter OsPT11 becomes undetectable [76].

### 5.3. Coordinated Roles of CSSP Core Components in Ca^2+^ Oscillations

SYMRK encodes a receptor-like kinase that acts as a co-receptor upstream of Ca^2+^ oscillations in the symbiotic signaling pathway; its dysfunction directly affects Ca^2+^ signaling initiation and AM symbiosis [77]. Both SYMRK and CASTOR mutations significantly reduce CO4-induced cytoplasmic Ca^2+^ peaks, maintain the first phase of cytoplasmic Ca^2+^ transients, but abolish the second phase of nuclear Ca^2+^ transients, suggesting their coordinated regulation of nuclear Ca^2+^ signals [46,72,78].

CASTOR and POLLUX both encode cation channels that act upstream of Ca^2+^ oscillations and are essential for Ca^2+^ spiking and nuclear Ca^2+^ signal generation. Mutations in either gene lead to Ca^2+^ oscillation defects, and mutants in *L. japonicus* and rice exhibit AM symbiosis defects: the fungus is restricted to forming hyphopodia and distorted hyphae on the root epidermis, failing to effectively invade the cortical layer, resulting in failure to form arbuscules and vesicles [46,72,79,80]. Loss of POLLUX function in rice also leads to loss of Blumenol accumulation, which is essential for cortical infection and arbuscule formation [37].

### 5.4. E3 Ubiquitin Ligase Regulation Within the CSSP

CERBERUS is a U-box protein with E3 ubiquitin ligase activity; its mutation reduces hyphal elongation along the root axis and decreases colonization levels [36,81]. LUMPY INFECTION (LIN) governs infection-thread polar growth during nodulation, yet its role in AM symbiosis remains unclear. Recent studies in *M. truncatula* reveal that LIN and its four homologs (LINL1–LINL4) function redundantly in both nodulation and AM symbiosis, and genetic disruption of these genes significantly impairs symbiotic colonization. The U-box, Armadillo-like, and WD40 domains are indispensable for LIN symbiotic activity. Both LIN and LINL1 exhibit U-box-dependent E3 ubiquitin ligase activity and interact with the core CSSP scaffold DELLA proteins through their U-box domains. Collectively, LIN/LINL family members represent redundant CSSP components that facilitate endosymbiotic microbial accommodation via U-box-mediated DELLA modulation [82].

## 6. Symbiotic Signaling Network

AM symbiosis establishment and homeostasis are governed by a sophisticated and interconnected regulatory network, covering upstream transcriptional control, plant endogenous post-transcriptional/protein fine-tuning, and fungal self-regulation coupled with plant immune balancing. These multi-dimensional modules act sequentially and synergistically to precisely control fungal infection, arbuscule morphogenesis, and the trade-off between symbiotic accommodation and immune defense.

At the transcriptional level, MYB transcription factors regulate AM symbiosis through distinct but interconnected pathways. OsMYBc regulates salt tolerance by targeting the potassium transporter gene *OsHKT1;1* [11]. The legume-specific MYB40 directly binds to flavonoid biosynthesis gene promoters and interacts with NSP2, promoting AM colonization under nutrient starvation; the *myb40* mutant exhibits an approximately 65% reduction in colonization rate, with normal arbuscule morphology and unaffected SL biosynthesis gene expression, indicating that the defect occurs at the early symbiotic signaling stage [83]. Notably, the flavonoid pathway plays a conserved positive role, as a gain-of-function mutation of CNGC15 enhances symbiosis and nutrient uptake by modulating flavonoid metabolism [52]. Together, these findings indicate that MYB factors and flavonoid metabolism form a regulatory network linking nutrient status, Ca^2+^ signaling, and transcriptional control of AM symbiosis.

Downstream of transcriptional regulation, multiple conserved post-transcriptional and signaling pathways fine-tune symbiotic intensity mainly through ROS, Ca^2+^/NO, and nutrient-dependent signaling. The CLE signaling pathway conservatively and negatively regulates AM symbiosis across multiple plant species. The MtCLE53-SUNN-RDN1 cascade restricts symbiotic progression, and mutation of this module enhances AMF colonization [84]. This regulatory function is conserved across plant species, as homologous CLE signaling components in tomato [85,86] and other crops similarly repress mycorrhization, representing a universal symbiotic constraint mechanism [85,86,87].

ROS homeostasis serves as a central regulatory hub, where small GTPase and pseudokinase proteins exert antagonistic effects to stabilize symbiosis. The small GTPase MtROP9 suppresses early fungal infection by promoting ROS accumulation, while its silencing facilitates symbiosis initiation [88]. By contrast, the CLE downstream pseudokinase MtCRN maintains ROS homeostasis to promote arbuscule development. The conserved function of CRN in *M. truncatula* [89] and maize [90] confirms the essential role of the CLE-ROP9-CRN regulatory axis in balancing symbiotic ROS responses.

miRNAs integrate nutrient and physiological signals to fine-tune symbiosis at the post-transcriptional level. miR399s act synergistically with SLs to negatively regulate mycorrhizal colonization under phosphate-sufficient conditions [18]. In tomato, the Sly-miR408b-SlBBP module modulates SOD activity and ROS scavenging, thereby dynamically adjusting mycorrhizal colonization to adapt internal plant physiological status [91]. Ca^2+^ and NO signaling act as core upstream sensing modules to support symbiosis activation and development. CDPK1 is involved in Ca^2+^ signaling and interacts with localized ROS production in epidermal cells during early fungal contact; its loss of function leads to significantly reduced colonization efficiency and arbuscule development [92]. MtAnn1 is a component of an ancient Ca^2+^ regulatory module, which is induced during AM symbiosis and is essential for normal arbuscule formation [93]. Additionally, PHYTOGB1 regulates AM symbiosis by modulating NO levels [94].

Complementing plant endogenous regulation, fungal self-regulation and plant immune reprogramming are essential prerequisites for successful symbiosis. The fungal transcription factor RiMsn2 controls fungal development and mycorrhization via the conserved RiHog1-RiMsn2-STREs module and impairs arbuscule formation and plant symbiotic adaptation [95]. At the plant immune interface, wall-associated kinases mediate the trade-off between symbiosis and defense: GhWAK13 suppresses immune responses to facilitate AM colonization, while GhWAK7A enhances immunity and restricts symbiosis. Cell wall damage signals such as oligogalacturonides further inhibit mycorrhization, confirming that active immune suppression is indispensable for symbiosis establishment [96].

## 7. Arbuscule Development and Maintenance

Arbuscule development and maintenance are the core functional stages of AM symbiosis, sustaining fungal accommodation and bidirectional nutrient exchange. This process is controlled by a sophisticated hierarchical regulatory network, in which the conserved RAM1–RAM2–WRI–FatM module acts as the central hub governing symbiosis-specific lipid biosynthesis, PAM assembly and arbuscule morphogenesis. Auxiliary transcription factors, vesicle trafficking components and receptor kinases fine-tune arbuscule development and stability, while senescence-related regulators mediate arbuscule turnover. These interconnected pathways collectively ensure the steady and sustainable operation of AM symbiosis.

### 7.1. Transcriptional Regulatory Network of Arbuscule Formation

The nuclear factor Y subunit NF-YC3 is a highly conserved positive regulator of AM symbiosis. In tomato and rice, NF-YC3 is strongly induced in arbuscule-containing cells, and its promoter is activated by the CYCLOPS-CCaMK complex. Knockdown or knockout of NF-YC3 leads to retarded or abnormally sized arbuscules, significantly reduced mycorrhizal colonization and arbuscule abundance, decreased phosphorus accumulation, and suppressed expression of AM marker genes (e.g., OsPT11) [97,98]. OsNF-YC3 is directly regulated by the phosphate starvation response master regulator OsPHRs and can form a heterotrimer with OsNF-YA11 and OsNF-YB11 to co-regulate symbiosis [98].

Downstream of NF-YC3 lies the central hub of the network RAM1, a core GRAS transcription factor highly conserved across multiple plant species, including *M. truncatula*, *S. lycopersicum*, *B. distachyon*, *O. sativa*, *L. japonicus*, *H. vulgare*, and *P. hybrida*. Loss of RAM1 function leads to failure or severe impairment of mycorrhizal colonization, defective appressorium formation, and blocked arbuscule development [11,12,99,100,101,102]. RAM1 interacts with another GRAS factor, RAD1, and together they regulate arbuscule development: RAD1 loss leads to slow intraradical colonization in *M. truncatula* or reduced arbuscule number and accelerated degeneration in *L. japonicus* [103,104]. MIG1 is a novel GRAS transcription factor that controls cortical cell expansion; its mutation leads to aberrant arbuscule morphology, reduced mature arbuscules, and increased malformed arbuscules [105].

WRI5s are AP2 transcription factors induced by AM fungi, regulating downstream target genes by binding to AW-box elements. WRI5a physically interacts with the bZIP factor SlHY5, and together they activate SlFatM-mediated fatty acid synthesis [106,107]. This regulatory module is conserved across plants: in *M. truncatula*, WRI5a acts downstream of CKL2 and participates in lipid supply by binding to the STR promoter [108]; loss of WRI5a/b in rice also leads to reduced mycorrhizal symbiosis [11]. ERF12 is a downstream negative regulator of WRI5a, suppressing arbuscule development and forming a negative feedback loop with WRI5a [109,110]. STR/STR1/STR2 are half-size ABCG transporters whose expression is regulated by the RAM1-WRI transcriptional module; mutations in these genes in *M. truncatula*, *L. japonicus*, and rice all lead to arbuscule formation defects [21,101,109,111]. SIP1 is an ARID-domain-containing transcription factor. Its long and short splice variants (SIP1L and SIP1S) can form oligomeric complexes. Mutation of SIP1 prevents fungal hyphae from entering cortical cells to form arbuscules, indicating that it specifically regulates fungal invasion and arbuscule establishment (Figure 4) [112].

### 7.2. Lipid Biosynthesis and Metabolic Regulation

Lipid biosynthesis is a key process for fungal colonization and PAM formation in AM symbiosis, regulated by a hierarchical network of upstream transcription factors. Multiple genes involved in the fatty acid biosynthesis pathway (e.g., MtPK, MtKAS I/II, MtKAR, MtENR I, MtFatM) are induced upon mycorrhizal fungal infection in *M. truncatula* and participate in AM symbiosis regulation [107].

SlFatM mediates the biosynthesis of 16-carbon fatty acids. Loss of SlFatM function or mutation of SlWRI5a/SlHY5 leads to impaired AM fungal colonization, defective PAM formation, and arbuscule collapse [113]. In the bryophyte *M. paleacea*, the WRINKLED (WRI) transcription factor directly regulates lipid metabolism in arbuscule-containing cells; CRISPR-Cas9 knockout of WRI results in arrested arbuscule development and fungal colonization restricted to intraradical hyphae, leading to termination of mutualistic symbiosis [114].

Downstream of transcriptional regulation, RAM1 directly activates its target gene RAM2, which encodes glycerol-3-phosphate acyltransferase (GPAT). RAM2 is essential for arbuscule formation and lipid export [106,115]; its mutation leads to reduced appressorium formation, aberrant arbuscules, and decreased fungal-specific lipid content, resulting in severely impaired colonization and abnormal hyphal branching after penetration in both *L. japonicus* and rice [101,116]. RAM1 also interacts with WRI transcription factors to co-regulate downstream genes required for arbuscule development [101]. FatM and RAM2 cooperatively fine-tune lipid synthesis to promote arbuscule development in *M. truncatula* [11].

CBX1, an AP2 transcription factor (a WRI1 homolog), co-regulates the expression of the phosphate transporter LjPT4 and the H^+^-ATPase LjHA1 by binding to CTTC and AW-box motifs [117] while also activating the lipid metabolism gene RAM2, thereby participating in fatty acid biosynthesis. The cortical-cell-membrane-localized CKL1 and CKL2 in *M. truncatula* are phosphorylation targets of DMI2 and LysM receptors and are essential for AM symbiosis. They initiate the lipid provisioning program by regulating transcription factor expression, a process that acts in concert with arbuscule branching and the RAM1 regulatory network [118].

### 7.3. PAM Formation and Vesicle Trafficking

Vesicle trafficking and PAM biogenesis form a core subnetwork supporting fungal intracellular accommodation and arbuscule development. The highly conserved VPY acts downstream of Ca^2+^ oscillations [119] and is indispensable for fungal intracellular colonization across multiple plant species [120,121,122,123]. Its homologous LjVPY2 also participates in mycorrhizal symbiosis [81]. VPY physically interacts and functionally coordinates with the AM-specific exocyst subunit EXO70I, which confers specialized exocytotic capacity to sustain PAM structural development [124]. A set of SNARE family proteins further fine-tune symbiotic membrane construction and arbuscule development: MtVAMP721d/e support normal arbuscule formation by regulating vesicle trafficking [125]. LjVTI12 modulates arbuscule maturation and senescence [126], and SYP132α specifically governs symbiotic exocytosis to prevent premature arbuscule collapse [127]. Moreover, multiple mycorrhiza-induced accessory proteins participate in symbiotic interface modulation. SCP1 promotes fungal development and normal arbuscule morphogenesis [128]. TSB maintains arbuscule branching and maturation via microtubule regulation [129], and SbtM1/3 facilitate intraradical hyphal growth and arbuscule colonization [130].

### 7.4. Arbuscule Maintenance and Receptor-like Kinase Regulation

Fine-tuning of arbuscule persistence and symbiotic homeostasis relies on a suite of conserved receptor-like kinases (RLKs) and cytoplasmic kinases, which act at the periarbuscular membrane to govern post-developmental symbiosis regulation. The PAM-localized serine/threonine kinases OsARK1 and OsARK2 function synergistically via a shared signaling pathway to sustain normal AM symbiosis; OsARK1 specifically facilitates vesicle formation and fungal colonization without altering arbuscule morphology [131], and the non-redundant activity of OsARK2 further consolidates this symbiotic regulatory process [132]. Beyond rice, the evolutionarily conserved KIN2-KIN3 RLK complex serves as a core positive regulator of symbiosis in legumes, with loss-of-function mutations (in *M. truncatula* and *L. japonicus*) leading to impaired mycorrhizal colonization and defective arbuscule development [133,134,135]. Mechanistically, KIN3 interacts physically with the cytoplasmic kinases AMK8 and AMK24 to form a complete RLK/RLCK regulatory cascade, and this module is transcriptionally controlled by CBX1 through AW-box-dependent binding; the conserved symbiotic function of this kinase module is further verified by the defective symbiotic phenotypes of rice OsRLCK171 (KIN3 ortholog) knockout plants [133]. Counterbalancing these symbiosis-promoting kinase modules, the RsbQ-like family protein SlDLK2 acts as a key negative regulator to prevent excessive symbiotic growth [136]. Together, these complementary positive and negative kinase signaling axes precisely balance arbuscule maintenance and symbiosis intensity, enabling robust and stable AM symbiosis.

### 7.5. Arbuscule Degeneration and Senescence

Arbuscule degeneration is an actively regulated program that balances nutrient exchange with plant resource allocation. Several transcriptional and hormonal regulators control the timing and extent of this process. MYB1 acts as a positive regulator of degeneration. MYB1 is involved in regulating arbuscule degeneration. Loss of MYB1 function leads to a significant reduction in the proportion of degenerated arbuscules and a marked increase in the proportion of medium and large arbuscules [137].

Sugar transport across the PAM is critical for arbuscule maintenance. MtSWEET1b is a sugar transporter localized to the PAM, primarily exhibiting sucrose efflux activity and participating in sucrose transport. Loss of its function leads to premature arbuscule collapse and a significant increase in the number of degenerated/collapsed arbuscules, indicating that MtSWEET1b plays a key role in arbuscule maintenance [138]. Similarly, mutation of the soybean SWEET6 also promotes arbuscule degeneration [139].

Beyond sugar transport, arbuscule degeneration is also tightly controlled by negative regulators such as LjRSDL. This C2H2 zinc finger protein regulates arbuscule degeneration through the hormone signal transduction pathway. Loss of LjRSDL blocks arbuscule degeneration, leading to increased mycorrhization rate and accumulation of large arbuscules; conversely, its overexpression accelerates arbuscule degeneration and suppresses mycorrhizal colonization and large arbuscule formation [140]. Thus, MYB1 and LjRSDL act antagonistically to fine-tune arbuscule lifespan.

Multiple metabolic enzymes influence degeneration through lipid remodeling, apocarotenoid synthesis, and glycosylation. GmPAP33 is a purple acid phosphatase involved in phospholipid hydrolysis during arbuscule degeneration. Its silencing blocks arbuscule degeneration and increases the proportion of small arbuscules [141]. MtGINT1 is a sphingolipid glycosyltransferase involved in the synthesis of specific glycosyl inositol phosphoryl ceramides (GIPCs) and is developmentally regulated in symbiotic tissues. Its silencing leads to impaired AM symbiosis, aberrant arbuscule development, and premature senescence [142]. MtDXS2 is a key enzyme in the MEP pathway, involved in the synthesis of apocarotenoids. Its mutation leads to reduced apocarotenoid accumulation, downregulation of marker genes such as MtPT4, and an increase in degenerated arbuscules accompanied by a decrease in mature arbuscules, indicating that the late stage of AM symbiosis is compromised [143]. SlUGT132 is a UDP-glycosyltransferase that negatively regulates AM development. Suppression of its function significantly promotes root colonization and arbuscule formation, suggesting that this enzyme maintains symbiotic homeostasis by limiting excessive symbiosis [144].

## 8. Symbiotic Nutrient Uptake

### 8.1. Transcriptional Regulation of Phosphate Uptake

The MYB transcription factors PHR1, PHR2, and PHR3 are core regulators of AM symbiosis. Loss of OsPHR1, OsPHR2, and OsPHR3 in rice leads to abnormal mycorrhizal colonization and impaired arbuscule development [11,145]. *M. truncatula* PHR2 regulates arbuscule development and phosphate uptake by activating P1BS-containing target genes [146]. Knockdown of MdPHR2 or MdARF6 in apple reduces mycorrhizal colonization and phosphate uptake. These two transcription factors synergistically activate MdPHT1;13 expression to promote phosphorus acquisition [147].

In rice, SPX proteins negatively regulate AM symbiosis by suppressing PHR activity. Because of high functional redundancy, only multigene knockout alleviates PHR inhibition, thereby enhancing mycorrhizal colonization and phosphate starvation responses through a derepression mechanism [11]. In contrast, Medicago SPX1 and SPX3 have more distinct functions: single mutants reduce mycorrhizal colonization and arbuscule abundance (suggesting positive regulation), whereas double mutants show both increased colonization and accelerated arbuscule degradation under low Pi, indicating that these proteins promote colonization while maintaining arbuscule stability [146,148].

### 8.2. Phosphate Transporters

The mycorrhiza-specific phosphate transporters of the PHT1 family are highly conserved across plant species and serve as core components for plant Pi acquisition in AM symbiosis. These proteins (e.g., LjPT3/LjPT4, OsPT11/OsPT13 in rice, SlPT3/SlPT4/SlPT5, MtPT4, AsPT1/AsPT4, and ZmPht1;6) mediate Pi uptake via the mycorrhizal pathway and maintain arbuscule structure and longevity. Loss of their function leads to reduced Pi uptake, decreased arbuscule number or premature degeneration, and in some cases (e.g., MtPT4) even termination of symbiosis [149,150,151,152,153,154,155]. Regulatorily, OsPT11 and OsPT13 are directly activated by the phosphate starvation regulator OsPHR2 via the P1BS element [151]. Furthermore, Pi transporters also respond to multiple nutrient stresses: SlPT3 is induced under combined Pi/Zn deficiency, participating in Pi transport, arbuscule development under Zn deficiency, and Fe homeostasis regulation [152]; ZmPht1;6 is involved in AM symbiosis and Pi/Zn uptake regulation [156]. This places the PHT1 transporters as the terminal effectors of the PSR network, translating transcriptional signals into nutrient flow.

### 8.3. Energy Supply

OsHA1, MtHA1, and SlHA8 are mycorrhiza-specific plasma membrane H^+^-ATPases primarily expressed in arbuscule-containing cells. They energize the symbiotic interface via proton pumping and are essential for arbuscule development and mycorrhizal phosphate uptake. Mutations lead to aberrant/degenerated arbuscules, reduced colonization, and severely impaired Pi uptake; SlHA8 also affects nitrogen uptake [157,158].

### 8.4. Nitrogen Uptake and Assimilation

Plants acquire nitrogen from AM fungi through both nitrate and ammonium transporters. GhGLN1.5 is a member of the glutamine synthetase family, catalyzing the conversion of inorganic nitrogen to glutamine and playing a key role in nitrogen assimilation. Silencing or knockout of this gene impairs AM symbiosis, resulting in reduced AM infection rates and decreased nitrogen uptake. The ethylene response factor GhWRI3 activates GhGLN1.5 expression via the AW-box, regulating nitrogen assimilation and AM symbiosis [159]. OsNPF4.5 is a mycorrhiza-induced nitrate transporter responsible for symbiotic nitrogen uptake; its mutation reduces nitrogen uptake by approximately 45% and significantly decreases arbuscule colonization [160]. ZmAMT3;1 is a high-affinity ammonium transporter localized to the PAM, mediating mycorrhizal nitrogen uptake; its RNAi silencing significantly reduces mycorrhiza-dependent nitrogen uptake, and its function does not depend on symbiosome formation [161].

### 8.5. Potassium and Zinc Uptake

SlHAK10 is a mycorrhiza-specific K^+^ transporter mediating mycorrhizal potassium uptake; enhanced potassium nutrition promotes fungal colonization [162]. MtZIP14 is a putative zinc transporter; its loss of function reduces fungal colonization, decreases vesicle and arbuscule colonization rates, and impairs AM colonization and AMF-mediated zinc uptake, particularly under low or sufficient Zn supply [163].

### 8.6. Carbon Supply and Symbiosis Maintenance

The plant provides carbon to the fungus in exchange. MtSucS1 (symbiotic sucrose synthase) plays a key role in arbuscule establishment and maintenance during AM symbiosis. Its knockdown significantly inhibits AM fungal colonization, impairs arbuscule development and maintenance, and leads to reduced plant phosphorus and nitrogen levels [164,165]. Carbon acquisition from the host plant is fundamental for maintaining AM symbiosis. MST2 is a plasma-membrane-localized high-affinity monosaccharide transporter primarily transporting glucose and is highly expressed during the fungal intraradical phase. Its loss of function reduces mycorrhization levels, decreases arbuscule number, and causes morphological abnormalities, indicating that MST2 is essential for functional AM symbiosis [166].

### 8.7. Hormonal and Peptide Signaling Regulation

Multiple plant hormones and peptide signals differentially govern AM symbiosis via cell-type-specific regulatory patterns. 

Gibberellin (GA) negatively modulates AM symbiosis by repressing cortical arbuscule formation and maturation via DELLA-dependent signaling. GA negatively regulates AM symbiosis in cortical cells by repressing arbuscule formation and maturation in a DELLA-dependent manner. MtDELLA1/2 and DELLA proteins in *L. japonicus* are essential for normal symbiotic development, and GA restricts cortical arbuscule formation and symbiotic efficiency by promoting DELLA protein degradation [167,168,169].

Auxin signaling is essential for the establishment and maintenance of AM symbiosis in tomato. The nuclear-localized SlARF6–SlIAA23 module acts antagonistically to regulate SL biosynthesis, thereby modulating mycorrhizal colonization and symbiotic phosphorus uptake [170]. OsARF12/25 positively promote AM symbiosis by regulating auxin-dependent phosphate homeostasis [11].

IAA, Ethylene, BR, JA and ABA mediate AM symbiosis progression with distinct regulatory functions. Mutation of IAA27 leads to a significantly reduced mycorrhizal colonization rate and arbuscule number [171]. Ethylene acts as a negative regulator of mycorrhizal colonization, and mutations or RNAi of pea EIN2 and rice OsEIL2 both lead to enhanced colonization [172]. In contrast, BR serves as a positive regulator of AM development; mutations of BR biosynthesis genes DX and LK markedly impair mycorrhization and arbuscule formation, and the sucrose transporter SlSUT2 participates in symbiotic modulation by interacting with BR signaling proteins [173]. LK, an ortholog of the BR synthesis gene DET2, exhibits a reduced colonization rate and arbuscule formation when mutated [172]. Similarly, JA is involved in AM-mediated plant physiological responses [174]; the JA biosynthesis mutant def-1 also affects water regulation under AM symbiosis [175]. ABA-deficient tomato mutants also exhibit impaired mycorrhiza formation [136]. The PP2AB’1 functions downstream of ABA and Ca^2+^ signaling. It regulates fungal infection in epidermal cells and cortical cell growth, thereby promoting AM symbiosis establishment [176].

The CEP2–CEPR1/CRA2 peptide module coordinates root architecture and AM symbiosis. AM symbiosis downregulates CEP2 expression to relieve its inhibition on auxin-mediated lateral root growth [177]. The CEP2 receptor CRA2 positively regulates mycorrhizal colonization but restricts lateral root development. Alteration of CEP2 signaling disrupts the balance between root development and symbiosis, confirming its core regulatory role [178].

## 9. Conclusions and Perspectives

Over the past two decades, more than 170 plant genes essential for AM symbiosis have been characterized, establishing a comprehensive molecular framework spanning fungal signal perception, symbiotic signaling transduction, and bidirectional nutrient exchange. Despite these advances, multiple key knowledge gaps still hinder an in-depth and systematic understanding of AM symbiosis regulatory mechanisms. First, the functional coordination between evolutionarily conserved core pathways and variable peripheral regulatory modules remains elusive. Second, the cross-species functional conservation and regulatory divergence of symbiotic genes are poorly clarified, particularly for numerous genes identified only in individual plant species. Third, most current mechanistic models are generalized from bulk tissue data, lacking resolution of cell-type-specific regulatory patterns and epigenetic and hormonal dynamics underlying layered symbiotic responses.

The SL biosynthesis and perception cascade constitutes a highly conserved core module governing AM symbiosis in land plants, with key genes, including RAM1, CCaMK, CYCLOPS, and CCD7/8, acting as indispensable molecular switches across diverse plant species. However, the molecular mechanism coordinating the conserved core and plastic peripheral pathways of the SL regulatory network remains poorly understood. Accumulating evidence indicates that NSP transcription factors broadly regulate the biosynthesis of symbiosis-related apocarotenoids, including not only SLs but also blumenols and other mycorrhiza-associated derivatives. These apocarotenoid metabolites serve as key compensatory signaling components that confer regulatory flexibility to symbiotic signaling, while distinct interspecific SL dependency further shapes the plastic characteristics of AM symbiosis. The precise regulatory logic and functional coordination of these flexible peripheral pathways still require further systematic exploration. Furthermore, the cross-species functional conservation of numerous genes currently reported only in single species (e.g., ADK1, AMK24, AMT3;1) lacks systematic validation. Genes involved in fundamental cellular processes such as Ca^2+^ signaling and protein phosphorylation are likely to exhibit higher evolutionary conservation, whereas those involved in nitrogen transport and hormone-specific signaling are more prone to functional divergence. Addressing these questions will directly impact the cross-species breeding application value of core conserved genes and the research priority and translational potential of single-species-reported genes in major cereal crops.

Current mechanistic insights into plant AM symbiosis are mostly derived from bulk tissue-level analyses, which mask intrinsic cell-specific symbiotic regulatory characteristics. AM symbiosis is a spatially precise process dependent on coordinated functional interactions between root epidermal and cortical cells, which is fine-tuned by upstream chromatin modification and local hormonal dynamics, rather than uniform whole-root responses. Cortical cells are the core functional site for arbuscule formation and PAM-mediated nutrient exchange, while epidermal cells are primarily responsible for initial fungal chitin perception and symbiosis initiation. Accumulating evidence suggests that most AM symbiosis-related genetic regulatory programs operate in both cell layers with distinct functional partitioning, yet these cell-type-specific regulatory differences remain poorly resolved in current studies. Future research may transcend conventional tissue-averaged detection methods, focus on cell-specific local hormonal patterns and epigenetic reprogramming, and explore how epigenetic regulation coordinates layer-specific cellular responses to modulate arbuscule development and symbiosis maintenance, which will facilitate a more systematic understanding of the complex AM symbiosis regulatory network.

Advanced molecular and omics technologies provide powerful tools to address the above challenges. CRISPR/Cas9 gene editing enables high-throughput functional validation and cross-species assessment of core and species-specific symbiotic genes in staple crops including rice, wheat, and maize, facilitating the screening of elite genetic targets for improved mycorrhizal colonization and nutrient uptake efficiency. Furthermore, the integration of spatial transcriptomics and cell-specific profiling technologies allows dynamic, single-cell resolution tracing of arbuscule formation, maturation, and degeneration, enabling precise dissection of cell-layer-specific symbiotic regulatory mechanisms. In-depth elucidation of AM symbiosis regulatory networks will not only enrich the mechanistic understanding of plant–microbe symbiosis but also provide valuable gene resources and theoretical support for breeding nutrient-efficient crop varieties, reducing agricultural phosphate input, and promoting sustainable green agriculture.

## Data Availability

All data presented in this review are derived from previously published peer-reviewed literature, with the corresponding references provided in the main text and supplementary tables. All data supporting the findings of this study are included in this published article and its supplementary information files (Table S1). No primary experimental datasets were generated or analyzed during this study.

## Supplementary Materials

The following supporting information can be downloaded at: https://www.mdpi.com/article/10.3390/genes17060686/s1, Table S1: List of plant genes that have been functionally validated to play a role in AM symbiosis and Protein sequences of characterized AM symbiosis-related genes and their homologs identified in nine species [179,180,181,182,183,184,185,186,187,188,189,190].
